# Type 1 diabetes and cardiovascular disease

**DOI:** 10.1186/1475-2840-12-156

**Published:** 2013-10-28

**Authors:** Oliver Schnell, Francesco Cappuccio, Stefano Genovese, Eberhard Standl, Paul Valensi, Antonio Ceriello

**Affiliations:** 1 Forschergruppe Diabetes e.V., Helmholtz Center Munich, Ingolstaedter Landstrasse 1, 85764 Munich-Neuherberg, Germany; 2 University of Warwick, Warwick, UK; 3 Department of Cardiovascular and Metabolic Diseases, Gruppo Multimedica, Sesto San Giovanni, Milan, Italy; 4 Service d’Endocrinologie-Diabétologie-Nutrition, Hôpital Jean Verdier, Bondy Cedex, France; 5 Insititut d'Investigacions Biomèdiques August Pi i Sunyer (IDIBAPS) and Centro de Investigación Biomédica en Red de Diabetes y Enfermedades Metabólicas Asociadas (CIBERDEM), Hospital Clínic Barcelona, Barcelona, Spain

**Keywords:** Type 1 diabetes, Cardiovascular disease

## Abstract

The presence of cardiovascular disease (CVD) in Type 1 diabetes largely impairs life expectancy. Hyperglycemia leading to an increase in oxidative stress is considered to be the key pathophysiological factor of both micro- and macrovascular complications. In Type 1 diabetes, the presence of coronary calcifications is also related to coronary artery disease. Cardiac autonomic neuropathy, which significantly impairs myocardial function and blood flow, also enhances cardiac abnormalities. Also hypoglycemic episodes are considered to adversely influence cardiac performance. Intensive insulin therapy has been demonstrated to reduce the occurrence and progression of both micro- and macrovascular complications. This has been evidenced by the Diabetes Control and Complications Trial (DCCT) / Epidemiology of Diabetes Interventions and Complications (EDIC) study. The concept of a metabolic memory emerged based on the results of the study, which established that intensified insulin therapy is the standard of treatment of Type 1 diabetes. Future therapies may also include glucagon-like peptide (GLP)-based treatment therapies. Pilot studies with GLP-1-analogues have been shown to reduce insulin requirements.

## Introduction

Over the past 40 years, a reduction in the mortality due to cardiovascular (CV) disease and coronary heart disease (CHD) by about 70% both in diabetic and non-diabetic patients has been observed [1]. The cause is presumed to be a substantial progress in CV risk factor management and interventional cardiology [1]. Furthermore, in patients with type 1 diabetes, a decrease in mortality and a remarkable improvement in life expectancy occurred during the past decades [2,3]. The comparison of two subcohorts of the Pittsburgh Epidemiology of Diabetes Complications study based on the period of diabetes diagnosis (1950–1964 vs. 1965–1980) found an increase in life expectancy by approximately 14 years [3]. Nevertheless, the overall risk of CVD for people with type 1 diabetes compared to people without diabetes is increased two- to threefold in men, and three- to fivefold in women. A significant increase in CVD mortality related to increasing HbA1c levels has been reported in type 1 diabetes [4].

The aim of this paper is to present an overview on epidemiologic and pathophysiologic aspects of the relation between type 1 diabetes and CVD. In addition, the management of risk factors, both with view on diagnostic and therapeutic approaches, is addressed.

## Epidemiology

In the EURODIAB IDDM Complications Study, including more than 3.200 patients with type 1 diabetes from 16 European countries, the prevalence of CVD was reported to be 9% in men and 10% in women, respectively [5]. Related to an increase in duration of diabetes and age, an increase from 6% in the age group of 15–29 years to 25% in the age group of 45–59 years, has been observed [5].

In type 1 diabetes as compared to type 2 diabetes, the relationship of hyperglycemia with microangiopathy as well as macroangiopathy seems to be more significant [6,7]. According to the results of a large Finnish database, CVD mortality in patients with type 1 diabetes aged from 45–64 years at baseline increases by about 50% with every 1% increase of glycated haemoglobin (HbA1c) [6].

In a population-based cohort of 879 individuals with type 1 diabetes from Wisconsin, hyperglycemia was associated with all-cause and cardiovascular mortality [8]. At baseline examination (1980–1982), patients were free of cardiovascular disease and end-stage renal disease. The patients were followed up until December 2001. The multivariable relative risks comparing the highest quartile of HbA1c (≥12.1%) with the lowest quartile (≤9.4%) were 2.42 (95% CI: 1.54, 3.82; p-trend = 0.0006) for all-cause mortality and 3.28 (95% CI: 1.77, 6.08; p-trend < 0.0001) for cardiovascular mortality [8]. This association was present among both sexes, and independent of duration of diabetes, smoking, hypertension, and proteinuria. The relation persisted in subgroup analyses by categories of diabetes duration, smoking, body mass index, proteinuria, and retinopathy [8].

In a Japanese study, which included type 1 diabetes, who were diagnosed at an age of <18 years between 1965 and 1979, CVD was identified as the leading cause of death in diabetes of more than 20 years of duration [9].

Recently, the long-term clinical outcomes and survival in patients with both young-onset type 2 and type 1 diabetes with a similar age of diagnosis were evaluated [10]. As compared with type 1, type 2 demonstrated to present to be associated with a more lethal phenotype and a higher mortality. Also diabetic complications were detected more frequently [10].

## Pathophysiology / Etiology

Long-term hyperglycemia, both in type 1 and type 2 diabetes, leads to microvascular and macrovascular complications [11]. Microvascular damage affects particularly the retina, kidneys, both the autonomic and peripheral nervous system, while the heart, brain, lower limbs, are affected by both micro- and macrovascular disorders [11].

### Oxidative stress

**Figure 1:**
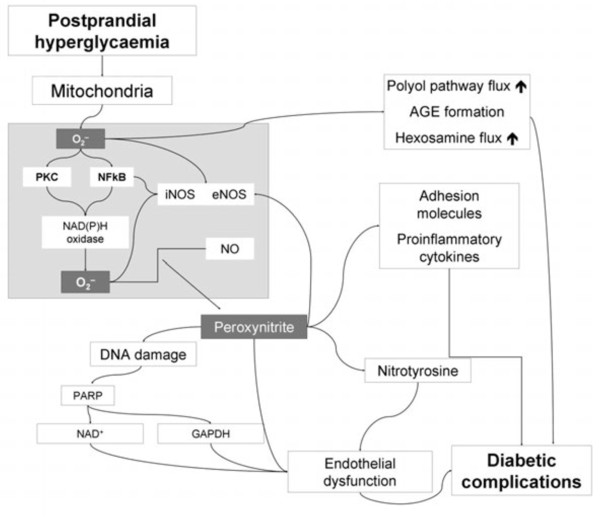
**Pathogenesis of diabetic complications: Hyperglycemia-induced overproduction of superoxide by the mitochondrial electron-transport chain is supposed to be the key element.** By activation of different pathways, the formation of the strong oxidant peroxynitrite is favoured, which in turn is damaging DNA. Through several intermediate steps, acute endothelial dysfunction, contributing to the genesis of diabetic complications, is triggered [13].

Hyperglycemia-induced overproduction of superoxide by the mitochondrial electron-transport chain is supposed to be the key element in the activation of all other pathways involved in the pathogenesis of diabetic complications (Figure 1) [12,13]. These include an increase in polyol pathway flux and advanced glycation end product formation, an activation of protein kinase C, and an increase in hexosamine pathway flux. Superoxide overproduction is accompanied by increased nitric oxide generation, due to an endothelial nitric oxide synthase (NOS) and inducible NOS uncoupled state. Thus, the formation of the strong oxidant peroxynitrite is favoured, which in turn is damaging deoxyribonucleic acid (DNA) [12,13].

Due to this DNA damage, a rapid activation of poly(adenosine diphosphate [ADP]-ribose) polymerase occurs, in turn depleting the intracellular concentration of its substrate nicotinamide adenine dinucleotide (NAD^+^), and slowing the rate of glycolysis, electron transport, and adenosintriphosphate (ATP) formation. In addition, the ADP-ribosylation of the glyceraldehyde 3-phosphate dehydrogenase (GAPDH) is stimulated. These processes result in acute endothelial dysfunction, which is contributing to the genesis of diabetic complications [12,13].

### Inflammation

Also an increase in inflammatory cytokines is supposed to contribute to plaque instability in patients with diabetes [14]. Several inflammatory markers including C-reactive protein, interleukin (IL)-6, IL-8, tumor necrosis factor (TNF)-α, and endothelin-1 are increased during hypoglycemia. The accumulation of inflammatory cytokines is assumed to cause endothelial injury and abnormalities in coagulation, resulting in increased risk for CV events [14].

### Hypercoagulability

The coagulation system is altered due to changes in clotting factor levels and/or activity. Plasma levels of procoagulant factors are increased while fibrinolytic capacity is decreased [15]. Hyperinsulinemia results in increased hepatic synthesis of prothrombotic factors such as fibrinogen and plasminogen activator inhibitor (PAI)-1, thereby creating a thrombotic milieu. Furthermore, diabetes causes quantitative modifications in clotting factors, including glycation and oxidation which also increase thrombosis risk [15].

### Autonomic neuropathy

Cardiac autonomic neuropathy (CAN) detected by standard tests is a common complication of type 1 diabetes. CAN prevalence is around 20% and increases with age and diabetes duration with an about 2% annual increase [16]. Poor glycemic control is a strong risk factor for CAN as supported by the EURODIAB study [17]. In the DCCT study intensive insulin treatment reduced the incidence of CAN by 53% compared to conventional therapy [18]. In the EDIC study, at the 13th-14th year after DCCT closeout, the prevalence and incidence of CAN remained significantly lower in the former intensive than in the former conventional group [19].

Several studies showed the predictive value of CAN on mortality [16] and that CAN is an independent predictor of mortality. CAN was reported to be a predictor of CV morbidity and mortality in type 1 diabetes [20]. Various CV disorders associated with CAN and resulting from vagal impairment and sympathetic predominance were shown mostly in type 2 diabetes and may account for the poor prognosis related to CAN [16]. Such disorders have been far less studied in patients with type 1 diabetes. In a study on patients with type 1 and type 2 diabetes, the prevalence of hypertension was shown to increase with CAN severity (from 3.6% in the patients without CAN to 36.4% in those with severe CAN), and CAN was an independent risk factor for hypertension [21]. This association suggests that vagosympathetic imbalance with a relative sympathetic overdrive may be involved in hypertension. In the Pittsburgh EDC study, CAN was associated with increased arterial stiffness 18 years later [22]. There is also strong evidence, based on studies in patients with type 1 or type 2 diabetes, that QT interval prolongation is an independent predictor of mortality, for all-cause and for cardiovascular deaths [16].

The balance of the activity of the autonomic nervous system is considered to play a key role for the performance of the diabetic heart [23]. Advanced single-photon emission computed tomography (SPECT) and positron emission tomography (PET) allow to directly and sensitively assess cardiac sympathetic innervation [24-29], coronary blood flow [30,31] and myocardial metabolism [32,33]. In long-term type 1 diabetes, myocardial blood flow response to sympathetic stimulation is significantly impaired.

Scintigraphically, cardiac sympathetic dysinnervation was identified in 77% of newly diagnosed metabolically stabilized type 1 diabetic patients [27]. The pattern of cardiac sympathetic dysinnervation of newly diagnosed type 1 diabetic patients is heterogeneous with a predominant affection of the posterior myocardial region [25,27]. More recent publications, however, emphasize that neither impairment of metabolic control nor the presence of CV denervation may be a prerequisite for the development of impaired vasodilatory reserve. Diastolic dysfunction occurring early in the course of type 1 diabetes has been reported to be associated with abnormal cardiac sympathetic function as assessed by cardiac sympathetic imaging [34,35].

Neuronal abnormalities are reported to progress with duration of diabetes [36]. In parallel, defects of cardiac sympathetic innervation are more enhanced in long-term than in newly diagnosed type 1 diabetic patients [25,27]. In patients with a long diabetes history, heterogeneity of cardiac sympathetic dysinnervation, characterized by a more advanced affection of the posterior myocardium in comparison to the anterior, lateral and septal myocardium has been observed [25]. In studies on small groups of patients with type 1 diabetes, frequent sympathetic dysinnervation and a predominance in the posterior myocardial region [37], and proximal sympathetic hyperinnervation of the heart [38] has been observed with PET.

Immunological factors against sympathetic ganglia have been reported to be associated with cardiac sympathetic dysfunction [39-45]. Autoantibodies against sympathetic ganglia have been found in 20-35% of type 1 diabetic patients [39,41,42]. The presence of autoantibodies against sympathetic ganglia has been shown to be associated with scintigraphically assessed cardiac sympathetic dysfunction [39,41] and electrocardiogram (ECG)-based abnormalities of heart rate variation [41]. Autoantibodies against sympathetic ganglia seem to be rather specific for cardioneuropathy of type 1 diabetic patients [41].

### Hypoglycemia

Additional hemodynamic changes have been reported to be associated with hypoglycemia [46]. An increase in heart rate and peripheral systolic blood pressure as well as a reduction in central blood pressure and peripheral arterial resistance (causing a widening of pulse pressure) has been described. Furthermore, an increase in myocardial contractility, stroke volume, and cardiac output has been observed [47]. In healthy people, arteries have been reported to become more elastic during hypoglycemia with a decline in wall stiffness [46]. In people with a longer history of type 1 diabetes, however, due to an enhanced arterial wall stiffness, hypoglycemia is followed by a less pronounced fall in central arterial pressure [46,48]. As a consequence, temporarily a markedly increase in the workload of the heart has to be assumed [46].

In the ECG, hypoglycemia has been found to elicit ST wave changes with lengthening of the QT interval [49] and cardiac repolarization [50]. Thereby, the risk for arrhythmia is assumed to be increased [46]. Related to hypoglycemia, various abnormal heart rhythms, including ventricular tachycardia and atrial fibrillation, have been observed. In conclusion, hypoglycemia has been found to potentially cause abnormal electrical activity in the heart and is assumed to can provoke sudden death [46].

An association between hypoglycemia and sudden death has been deteced by different investigators [51-56]. In line with the hypothesis an autopsy study demonstrated, that sudden unexpected deaths were four times more frequent in type 1 diabetic patients than in nondiabetic people [56].

## CV risk

### Risk factors for microvascular complications

The risk of microvascular complications is influenced by several factors, such as puberty, blood pressure, dyslipidemia, gender, diabetes duration, smoking and lifestyle [57-59]. Poor metabolic control was identified as an important factor contributing to microvascular complications [60,61]. In addition, familial risk factors related to all microvascular complications of type 1 diabetes have been reported [62]. A study performed in type 1 diabetic patients (onset age < 30 years) among 6,707 families revealed a significantly increased risk of retinopathy (odds ratio 9.9; CI 5.6–17.7, P < 0.001), nephropathy (6.2; CI 2.9–13.2, P < 0.001) and neuropathy (2.2; CI 1.0–5.2; P < 0.05) in type 1 diabetic siblings of patients diagnosed with those complications [62].

In an analysis of 572 type 1 diabetic participants of the Pittsburgh EDC Study (mean follow-up: 15 years), baseline HbA1c was an independent risk factor for fatal CAD, along with duration of diabetes and albuminuria [63]. Baseline lower insulin dose, however, was strongly predictive for non-fatal CAD, as was lower renal function, higher diastolic blood pressure, and lipids [63].

In patients with diabetes onset at age 5–14 years, a higher risk for complications (retinopathy, nephropathy, and neuropathy) has been found as compared to patients diagnosed either at a very young age or after puberty [62]. In adolescents with type 1 diabetes, an elevated blood pressure or BMI [64-66], dyslipidemia and smoking [67-69] are associated with an elevated risk of incipient nephropathy, early retinopathy and peripheral neuropathy.

With the onset of diabetic nephropathy, a dramatic increase in the risk for CAD has to be assumed. After 20 years with diabetes, up to 29% of patients with childhood-onset of type 1 diabetes and nephropathy will have CAD compared to only 2–3% in similar patients without nephropathy [70]. In addition to traditional cardiovascular disease risk factors, elevated mean HbA1c and macroalbuminuria are significantly associated with alterations in left ventricular structure and function evaluated by cardiac magnetic resonance imaging (MRI) [71].

In observational studies the relationship between blood pressure and the progression of chronic kidney disease (CKD) and incident end-stage renal disease (ESRD) is direct and progressive in diabetes [72]. However, most of the evidence is in type 2 diabetes, high blood pressure is a common feature also of type 1 diabetes and an increase in blood pressure in type 1 diabetes increases the risk of nephropathy [73,74]. Masked hypertension is not infrequent [72]. In people with type 1 diabetes an increase in systolic blood pressure, particularly at night, precedes the development of microalbiminuria [75]. It has been argued that, unlike in type 2 diabetes, in people with type 1 diabetes hypertension develops often after the establishment of microalbuminuria. Hence, monitoring blood pressure throughout the 24 hours in type 1 diabetes may be a useful diagnostic procedure.

In the DCCT/EDIC study, during a 15.8-year median follow-up, 630 of 1441 participants developed hypertension [76]. Intensive therapy during the DCCT reduced the risk of incident hypertension by 24% during EDIC study follow-up. A higher HbA1c level, measured at baseline or during follow-up, was associated with increased risk for incident hypertension. Older age, male sex, family history of hypertension, greater baseline body mass index, weight gain, and greater albumin excretion rate were independently associated with increased risk of hypertension. These data show that hyperglycemia is a risk factor for incident hypertension in type 1 diabetes and that intensive insulin therapy reduces the long-term risk of developing hypertension.

In a recently published Brazilian study on approximately 1,300 patients with type 1 diabetes, however, body size and blood pressure were not correlated to lipid levels and glycemic control [77]. Correlation of serum lipids with HbA1c was shown to be heterogeneous across the spectrum of glycemic control. Several pathophysiological factors were suggested based on the HbA1c-level. These results, therefore, do not support a unified explanation for cardiovascular risk in type 1 diabetes [77].

### Cardiovascular risk markers

As demonstrated in 144 participants of the Pittsburgh EDC Study, pulse wave analysis (PWA) may contribute to assessment of CV risk in patients with type 1 diabetes [78]. Arterial stiffness index, augmentation index, augmentation pressure, subendocardial viability ratio (serving as an estimate of myocardial perfusion), electron beam computed tomography-measured coronary artery calcification (CAC) and ankle-brachial index (ABI) were determined. In the analysis of cross-sectional associations, greater augmentation pressure was independently associated with prevalent CAD and estimated myocardial perfusion with low ABI (<0.90) [78].

In the DCCT/EDIC study the stiffness/distensibility of the ascending thoracic aorta was measured with magnetic resonance imaging in 879 patients [79]. After adjusting for gender and cohort, aortic distensibility was lower with increasing age, mean systolic blood pressure, LDL cholesterol, and HbA1c measured over an average of 22 years. Patients with macroalbuminuria had 25% lower aortic distensibility compared with those without, and lower distensibility also was associated with greater ratio of left ventricular mass to volume. This data stand in favour of strong adverse effects of hypertension, chronic hyperglycemia and macroalbuminuria on aortic stiffness in type 1 diabetes.

After 15 years additional follow-up in EDIC, left ventricular indices were measured by cardiac magnetic resonance imaging in 1017 of the 1371 members of the DCCT cohort [80]. Mean DCCT/EDIC HbA1c over time was associated with end diastolic volume, stroke volume, cardiac output, left ventricular mass, LV mass/EDV, and aortic distensibility. These associations persisted after adjustment for CVD risk factors. Thus, cardiac function and remodeling in the EDIC cohort was associated with prior glycemic exposure (glycemic memory).

As part of the EDIC study, 1229 patients with type 1 diabetes underwent ultrasonography of the internal and common carotid arteries in 1994–1996 and again in 1998–2000 [81]. At year 1 of the EDIC study, the carotid intima-media thickness (IMT) was similar to that in an age- and sex-matched nondiabetic population. After six years, the IMT was significantly greater in the diabetic patients than in the controls. The mean IMT progression was significantly less in the group that had received intensive therapy during the DCCT than in the group that had received conventional therapy after adjustment for other risk factors. IMT progression was associated with age, and the EDIC base-line systolic blood pressure, smoking, the LDL/HDL ratio, and urinary albumin excretion rate and with the mean HbA1c value during the mean duration of the DCCT. Thus, intensive therapy during the DCCT resulted in decreased progression of IMT six years after the end of the trial, which again stands in favour of the effect of glycemic memory.

As found by the 10-year follow-up examination of the Pittsburgh Epidemiology of Diabetes Complications (EDC) Study cohort, CAC is related to clinical coronary artery disease (CAD) independent of other risk factors [82]. This association, however, was stronger in men than in women [82]. In a cohort of patients with type 1 diabetes (aged 22–50 years), progression of CAC, as identified by electron beam computed tomography (EBCT), was strongly associated with suboptimal glycemic control (HbA1c >7.5%) [83].

In a study assessing CAC with multislice spiral computed tomography (MSCT), nearly one third of asymptomatic long-term type 1 diabetic patients presented with coronary calcifications [84]. In patients with coronary calcifications, both cardiac autonomic neuropathy and retinopathy were detected more frequently than in those without (64% vs. 29%, p < 0.02; 59% vs. 31%; p < 0.02). Additionally, duration of diabetes was longer in patients with than without coronary calcification [84].

In a small cohort of adolescent, non-obese type 1 diabetic patients, an increased carotid intima-media thickness was found to be associated with insulin resistance. A causal relationship, however, cannot be concluded [85]. According to a prospective longitudinal study in children and adolescents with type 1 diabetes, systolic blood pressure and body mass index are related to carotid intima-media thickness increment. Control of these risk factors is supposed to contribute to prevention of carotid intima-media thickness progression [86].

In patients with long-term duration type 1 diabetes, sexual dysfunction was demonstrated to be independently associated with CVD and to potentially predict CVD [87].

Results on the predictive value of plasminogen activator inhibitor-1 (PAI-1) are inconsistent. One study found PAI-1 levels to be independently related to CAC in younger (< 45 years) patients with type 1 diabetes [88]. According to another analysis, neither PAI-1 nor tPA-PAI-1 is an independent predictor of CAD [89].

## Diagnosis / Screening

In type 1 diabetes, hypertension is often the result of nephropathy. Blood pressure measurement at every routine visit is recommended [90]. In most adult patients with diabetes, a fasting lipid profile at least once a year is recommended [90]. Low-risk lipid values (LDL cholesterol < 100 mg/dL, HDL > 50 mg/dL, triglycerides < 150 mg/dL) provided, assessment may be repeated biannually [90].

In type 1 diabetic patients with diabetes duration ≥ 5 years, the screening for nephropathy should include an annual assessment of urine albumin excretion [90]. Irrespective of the degree of urine albumin excretion, in all adults with diabetes serum creatinine should be measured at least annually [90]. The creatinine value is useful for estimation of glomerular filtration rate (GFR) [90]. In children and adolescent patients, annual screening both for nephropathy and retinopathy is recommended to start at age 11 years in case of 2 years diabetes duration and at age 9 years with 5 years duration, respectively [91].

Screening for signs and symptoms of CV autonomic neuropathy should be started 5 years after the diagnosis of type 1 diabetes [16,90]. CV reflex tests are the gold standard in clinical autonomic testing. The most widely used tests assessing cardiac parasympathetic function are based on the time-domain heart rate response to deep breathing, Valsalva maneuver, and postural change. Age is a strong modulator of these tests and needs to be considered when interpreting the results. CV sympathetic function is assessed by measuring the blood pressure response to standing [92].

PWA may contribute to assessment of CV risk in patients with type 1 diabetes [78]. In a study, greater augmentation pressure was independently associated with prevalent CAD and estimated myocardial perfusion with low ABI (<0.90) [78]. Screening patients for silent myocardial ischemia is controversial but seems reasonable in very high risk patients, in particular in those with long duration of diabetes and proteinuria or evidence of peripheral artery disease, and in those who wish to start a vigorous exercise program. Measurement of CAC score may be suggested as a first line investigation, leading to a stress test if the score is higher than 400 [93,94].

## Treatment

### Gycemic control

Intensive insulin therapy has been strongly demonstrated to reduce the onset as well as progression of all diabetes-related microvascular complications [58,60,95-98]. The Diabetes Control and Complications Trial (DCCT)/ Epidemiology of Diabetes Interventions and Complications (EDIC) study found in approximately 1,200 patients with type 1 diabetes a relative CVD risk reduction of 40%, adjusted for other risk factors including albuminuria, when comparing intensive vs. standard treatment (mean HbA1c 7.4% vs. 9.1%) for 11 years [58]. In adolescent patients, intensive treatment (HbA1c 8.1%) as compared to conventional treatment (HbA1c 9.8%) has been shown to reduce the risk and progression of background retinopathy by 53%, clinical neuropathy by 60%, and microalbuminuria by 54% [60]. A prolonged effect of early intensive approaches was also seen in a four year follow-up of intensively treated adolescent type 1 diabetic patients [99].

Several studies confirmed the association between poor glycemic control and an increasing risk for nephropathy [100-102], retinopathy [95,103,104], and neuropathy [105-109]. A large proportion of patients, however, fails to achieve glycemic targets [110-112].

### GLP-1 based treatment as an add-on to insulin

Due to their action on insulin secretion and glucose regulation, glucagone-like peptide (GLP)-1 based treatment approaches have been established in the treatment of type 2 diabetes. Based on in vitro and animal studies, GLP-1 based drugs have been assumed to additionally may be effective in preserving and even expanding the beta cell mass [113]. In a small study on 15 patients with newly detected type 1 diabetes, the addition of exenatide at onset of diabetes has been shown to decrease insulin requirement [113]. Three groups of patients have been formed: group1 (insulin alone), group 2 (insulin and exenatide), and group 3 (insulin and sitagliptin).

After one year, a decrease in insulin requirement of 16.7 ± 12.5, 39.8 ± 17.2, 21.2 ± 9.6 units in groups 1, 2 and 3 respectively (P = 0.0431) was detected. A mean stimulated c peptide secretion of 0.34 ± 0.12, 0.45 ± 0.34, 0.44 ± 0.5 ng/mL was found (P = 0.8656). The maximum percentage preservation in c peptide was observed in the patients of group 2 [113]. Of relevance is the evidence that GLP-1 can protect type 1 diabetic patients by both acute hyperglycemia or hypoglycaemia induced endothelial dysfunction, oxidative stress and inflammation [114].

### Approaches beyond glycemic control

In contrast to DCCT/EDIC, some trials did not confirm the association between glycemic control and CVD risk [115-117]. Discrepancies are suggested to be based on differences between the study populations [118]. With view on prevention and treatment of CAD, it is recommended to focus not only on glycemic control [118]. Traditional risk factors such as albuminuria, the metabolic syndrome, and inflammatory markers should also be addressed [118].

International guidelines recommend lowering blood pressure in diabetes to prevent macro- and micro-vascular outcomes. However, most evidence from randomized clinical trials refers to type 2 diabetes. A goal of blood pressure <130 and <80 mmHg has been recommended [90,119]. The most recent recommendations of the American Diabetes Association (ADA) set a blood pressure goal of <140/ <80 mmHg for persons with diabetes and hypertension; lower targets (such as <130 mm Hg) may be appropriate in patients if the specific target can be achieved without an additional burden of treatment [90].

Pharmacological therapy in people with diabetes and hypertension should be with a regimen that includes either an angiotensin converting enzyme (ACE)-inhibitor or an angiotensin-receptor blocker (ARB) [90]. Generally, two or more drugs are required to achieve blood pressure targets in diabetics. Preferred combinations are either ACE-inhibitors or ARBs (not together) with a calcium-channel blocker or a diuretic. For the latter, both thiazide [90] and, more recently, thiazide-like diuretics [120] are recommended.

However, more recent appraisal of the evidence indicates lack of evidence to support systolic blood pressure targets <130 mmHg and suggests optimal diastolic blood pressure between 80 and 85 mmHg [72].

In studies among patients with diabetes, regular physical activity has been demonstrated to reduce CVD-related and total mortality [11]. Early treatment of hypertension has been reported to prevent end-stage kidney disease in patients with type 1 diabetes [121]. Angiotensin-converting enzyme (ACE) inhibitors have been demonstrated to be effective and safe in in reducing microalbuminuria [122]. In adolescent patients with persistent microalbuminuria, the use of ACE inhibitors [91,100,123] or angiotensin II receptor blockers [91] is recommended to prevent the progression to macroalbuminuria. Furthermore, in order to reduce progression of microalbuminuria, cessation of smoking is strongly recommended [117,124,125].

Lifestyle modification is recommended for the improvement of lipid profile. In diabetic patients with overt CVD, statins should be added irrespective of lipid levels [90]. Statin therapy is also recommended in diabetic patients without CVD aged > 40 years and ≥ 1 other CVD risk factor (family history of CVD, hypertension, smoking, dyslipidemia, albuminuria) [90]. In patients with lower CV risk, statin therapy should be considered if LDL cholesterol remains above 100 mg/dL [90]. In diabetic patients without overt CVD, the goal for LDL cholesterol is 100 mg/dL (2.6 mmol/L). In patients with overt CVD, a LDL cholesterol goal of 70 mg/dL (1.8 mmol/L), using a high dose of a statin, may be considered [90].

## Discussion / Outlook

Despite of a remarkable improvement in life expectancy, type 1 diabetic patients are confronted with an increased risk of CV mortality, an evidence often underrecognized. Further improvement may base on consequent risk assessment and management of risk factors. Estimation of CV risk including the role of surrogate markers deserves more attention in the future. Currently, optimized glycemic control is considered to be the most promising approach. Recent research on a small group of patients suggests the addition of exenatide at onset of diabetes to decrease insulin requirement. These results, however, need to be confirmed by further investigation on larger groups of patients.

## Competing interests

The authors declare that they have no competing interests.

## Authors’ contributions

All authors contributed to conception and design, drafting the article, revising the article critically, final approval of the version to be published.
